# Detecting Community Structures in Networks by Label Propagation with Prediction of Percolation Transition

**DOI:** 10.1155/2014/148686

**Published:** 2014-07-07

**Authors:** Aiping Zhang, Guang Ren, Yejin Lin, Baozhu Jia, Hui Cao, Jundong Zhang, Shubin Zhang

**Affiliations:** ^1^College of Marine Engineering, Dalian Maritime University, Dalian 116026, China; ^2^Department of Architectural Engineering, Jilin Province Economic Management Cadre College, Changchun 130012, China

## Abstract

Though label propagation algorithm (LPA) is one of the fastest algorithms for community detection in complex networks, the problem of trivial solutions frequently occurring in the algorithm affects its performance. We propose a label propagation algorithm with prediction of percolation transition (LPAp). After analyzing the reason for multiple solutions of LPA, by transforming the process of community detection into network construction process, a trivial solution in label propagation is considered as a giant component in the percolation transition. We add a prediction process of percolation transition in label propagation to delay the occurrence of trivial solutions, which makes small communities easier to be found. We also give an incomplete update condition which considers both neighbor purity and the contribution of small degree vertices to community detection to reduce the computation time of LPAp. Numerical tests are conducted. Experimental results on synthetic networks and real-world networks show that the LPAp is more accurate, more sensitive to small community, and has the ability to identify a single community structure. Moreover, LPAp with the incomplete update process can use less computation time than LPA, nearly without modularity loss.

## 1. Introduction

A complex system from nature, society, or any other field can usually be represented as a complex network: a structure with vertices and edges between vertices [1–9]. Community structure is a very important property of complex networks, which is generally described as a group of vertices: the edges in a group are denser, and the edges between groups are sparser [2]. Even in weighted networks, though they may consist of differentiated mass of connected vertices, there may still exist as distinct communities groups of vertices within which the edges are denser and between which the edges are sparser [3]. More and more algorithms are proposed and developed to detect the community structure, especially in recent years, such as Girvan-Newman algorithm (GN) [2], spectral clustering [4], spin-glass model [5], the algorithm proposed by Clauset, Newman, and Moore (CNM) [6, 7], partition method using integrating attributes of vertices [8], and extremal optimization [9].

The fastest one in these algorithms for community detection is label propagation algorithm [10]. Zhang et al. generalize LPA to weighted networks by calculating the probability of every label (WLPA) [11]. In a network, the two vertices neighbors are called neighbors if they are connected by an edge. Suppose that every vertex has a label to denote the community it belongs to. If a vertex's most neighbors have the same label, the label is called the maximal label of the vertex. The LPA can be described as follows. Initially, assign each vertex a unique initial label. At each iterative step, each vertex updates its label to its current maximal label in a random order. When there are multiple maximal labels, the vertex will randomly pick one of them as its label. If the label of every vertex in the network is its maximal label, the algorithm will be terminated. This is LPA's asynchronous version. We will not consider the synchronous version because of the potential label oscillations as discussed in [10]. LPA has three remarkable features. The first feature of LPA is its near-linear time complexity. For a network of *n* vertices and *m* edges, the time complexity of LPA is *O*(*m* + *n*). The second feature is that its capability of community detection is scale-independent. It is not affected by the resolution limit as the methods based on modularity. The third famous feature of LPA is its randomness, which includes random initial label, random order of label update, and randomly picking one of the maximal labels as the vertex label when the maximal label is not unique. Due to the randomness in label propagation, when LPA is used to detect the communities in a network, any information about this network except its vertices and edges need not be provided, and then the multiple community structures usually are obtained. On one hand, the randomness can make those community structures which are hard to be found in other fixed algorithms be detected easily by LPA; on the other hand, the small communities are likely to be missed, and trivial solutions are more likely to be obtained [12]. To detect small communities, LPA must be run many times, which makes its first two advantages less apparent.

After Newman introduced the modularity to measure the quality of network division, Barber and Clark proposed a modularity-specialized LPA (LPAm) to constrain the label propagation process [13–15]. Though it is prone to get stuck in poor local maximum of modularity, LPAm is still a near-linear time algorithm [16]. Liu et al. introduce an advanced modularity-specialized label propagation algorithm by combining LPAm with multistep greedy agglomerative algorithm, which is called LPAm+ [16, 17]. LPAm+ does not cost near-linear time any more, but it is more stable than LPAm. However, due to the usage of modularity, the capability of the two algorithms will be affected by resolution limit, though modularity is as a key fitness indicator [18, 19]. The obtained community structure using LPA-*δ* proposed by Leung et al. is still scale-independent because the algorithm does not involve modularity optimization [12]. The algorithm encourages a stronger local community by adding a decreasing score assignment for each label in label propagation process to deter the occurrence of trivial solution successfully. Leung et al. provide an idea to save the running time by avoiding label update of those vertices with high neighbor purity [12]. It does do well in saving time while not doing well in accuracy because the neighbor purity condition ignores contribution of the small degree vertices to community detection.

In this paper, we propose LPAp by adding the prediction process of percolation transition and introduce the incomplete condition in label propagation process to reduce the running time. According to the principle of percolation transition, when there are multiple maximal labels, we predict the sizes of the new communities and let the vertex choose the label which can make the smallest community as its label. LPAp is applicable to weighted networks. The incomplete condition considers both the neighbor purity and the vertex degree. Then we apply it to community detection on synthetic networks and real-world networks. As we will show, the LPAp is more accurate and can be faster than the original algorithm.

## 2. Problem Description

An unweighted network of *n* vertices can be described by an *n* × *n* adjacency matrix *A*, whose element is

(1) Aij={1if  i  and  j  are  connected,0otherwise.

For a weighted and undirected network of *n* vertices and *m* edges, denote the weight of connection between *i* and *j* by *w*_*ij*_; then the element of its *n* × *n* adjacency matrix *A* ^*w*^ is given by

(2) Aijw={wijif  i  and  j  are  connected,0otherwise.

An unweighted network can be considered as a weighted network, and the weight of every edge in this network is 1. For a weighted network, the degree *k*_*i*_ of a vertex *i* can be given by

(3) $$\begin{array}{c} k_{i}=\overset{n}{\underset{j=1}{\sum }}A_{ij}^{w}, \end{array}$$

and the number *m* of edges can be represented by

(4) $$\begin{array}{c} m=\frac{1}{2}\overset{n}{\underset{i=1}{\sum }}k_{i}. \end{array}$$

In most cases, the groups of vertices in a network identified by a community detection algorithm are assumed to be communities irrespective of whether these groups satisfy a specific definition or not as mentioned in [6, 8]. Then the quality of network division is measured by modularity, whose value is in [0, 1] [13, 14]. For a given network, the larger the modularity obtained by a method for network division, the better the quality of this division.

Suppose a network is unweighted, and it is divided into *N* communities. The number of edges in community *r* can be given by

(5) $$\begin{array}{c} I_{r}=\underset{i\in r,j\in r}{\sum }A_{ij}, \end{array}$$

and the sum over all degrees of vertices in community *r* can be given by

(6) $$\begin{array}{c} D_{r}=\underset{i\in r}{\sum }k_{i}. \end{array}$$

The modularity can be calculated according to the sum of contributions to every community [3, 16]:

(7) $$\begin{array}{c} Q=\overset{N}{\underset{r=1}{\sum }}\left(\frac{I_{r}}{m}-{\left(\frac{D_{r}}{2m}\right)}^{2}\right). \end{array}$$

When the modularity is used to measure the quality of division for a weighted network, *I*_*r*_ should indicate the sum over all weights of edges in community *r*. We can recalculate it as

(8) $$\begin{array}{c} I_{r}=\underset{i\in r,j\in r}{\sum }A_{ij}^{w}, \end{array}$$

so that (7) can be applied for weighted networks [3].

Denote the label of vertex *j* by *l*_*j*_. In LPA, after random initial label association, for every vertex *i*, its label updates in a random order according to the following rule:

(9) $$\begin{array}{c} l_{i}^{\text{new}}=\underset{l}{\arg\;\max}\left(\overset{n}{\underset{j=1}{\sum }}A_{ij}\delta \left(l_{j},l\right)\right), \end{array}$$

where *l*_*i*_^new^ indicates *i*'s new label and *δ*(*i*, *j*) is the Kronecker delta.

The labels of all the vertices are updated iteratively until every vertex satisfies the following condition:

(10) $$\begin{array}{c} l_{i}=\underset{l}{\arg\;\max}\left(\overset{n}{\underset{j=1}{\sum }}A_{ij}\delta \left(l_{j},l\right)\right), \end{array}$$

where *l*_*j*_ indicates *i*'s current label.

When *A*_*ij*_ is replaced by *A*_*ij*_^*w*^ in (9) and (10), LPA becomes WLPA which is applicable for weighted networks.

Using LPA, a trivial solution is often obtained; that is, the whole network is divided into a single community structure. If a single community evolves to dominate the whole network in community detection process, the community is called “monster community,” which eats small community structures [12]. If monster community occurs in label propagation process, a trivial solution is obtained in all probabilities.

Sometimes the trivial solution indicates a network's indivisibility. There is a kind of homogenous network whose edges are so dense that we cannot subdivide the network, and we call it a single community structure though there is no community structure [14]. We cannot divide it into some groups of vertices, and the edges in each group are denser than the edges in the network. An example of the single community network is a complete graph, any two vertices of which are neighbors [25]. A monster community obtained by LPA is meaningful in this case. The network should not be subdivided.

In most cases, the trivial solution is meaningless for community detection; there are usually multiple communities in the divided network. For example, ER random model is a kind of typical random networks proposed by Erdös-Rényì in 1965 [26]. For an ER random model, whose average degree is 4, when the network size is between 100 and 10000, LPA always identifies all the vertices in its giant connected component as a single community in [10]. ER random models are considered as the homogenous networks with no community [10]. The result of LPA seems to be right. But in fact ER random models whose edges are dense tend to have a handful of large communities, while ER random models whose edges are sparse tend to have many small communities [5]. LPA does not find the real community structures in ER random models whose edges are sparse.

**Figure 1:**
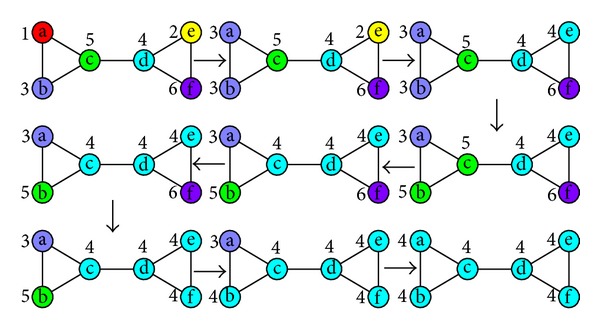
An illustration of monster community occurrence.

For a network, when one of its real communities is not much smaller than anyone of other real communities, a monster community can still occur and eat the real community in label propagation process. Though the sizes of real communities are equal, the monster community could occur in label propagation process. In Figure 1 we give a simple example of “monster community” occurrence in label propagation process. For its generalization we give every edge the same weight 1. A letter in a circle indicates a vertex, and the number near the circle indicates the vertex's label.

The network in Figure 1 is constructed by two equal-size communities intuitively. According to (7), the modularity of two-community division should be 0.36. Label update sequences of vertices are a-e-b-c-d-f at iteration 1 and b-a-f-c-e-d at iteration 2. After vertex a updates its label to 4 at iteration 2, every vertex's label in the network is its current maximal label. The termination condition of label propagation algorithm is satisfied, and the algorithm is terminated. It is noteworthy that vertex b chooses 4 as its label at iteration 2, which leads all the vertices to choose 4 as their labels at last. Then the modularity can be calculated by

(11) $$\begin{array}{c} Q_{s}=\frac{m}{m}-{\left(\frac{\sum_{i=1}^{n}k_{i}}{2m}\right)}^{2}=1-{\left(\frac{2m}{2m}\right)}^{2}=0. \end{array}$$

The modularity value of this division is the minimum, so it is not a good division. But it is reasonable; the termination condition of LPA is satisfied. Due to monster community, the right communities cannot be detected in label propagation process. It occurs in label propagation process. We can infer that the probability of monster community occurrence can be decreased if we make a change on label propagation process, so that the probability that trivial solutions are obtained can be decreased.

There are two reasons why the community structures obtained by LPA are not unique: the termination condition is a condition, not a measure to be optimized, and the other reason is its randomness. As mentioned in Section 1, the assignment of the initial label is random, the sequence of label propagation at each iterative step is random, and when there are multiple labels the most neighbors of a vertex have, the vertex will pick one randomly. If we change the termination condition into a measure or change the sequence of label propagation into a fixed sequence, the effect will be global. In other words, the LPA may lose the capability of detecting multiple solutions. Thus we only make a change in locality to keep the precious feature of label propagation process. Without changing the termination condition and the sequence of label propagation, we will give a priority list when there are multiple maximal labels in the next section.

## 3. Prediction Process of Percolation Transition

When there is a path between two vertices, we call the two vertices “connected.” Two connected vertices can be connected by one edge directly or by some other vertices and edges indirectly. If any two vertices in a graph are connected, the graph is called connected graph. Achlioptas et al. consider percolation transition phenomenon in random network construction process, which is known as the emergence of a giant connected component, and point out that “percolation transitions in random networks can be discontinuous” [26]. They start with *n* isolated vertices and then add edges one by one. Which edge will be added is decided by selection rule, and different rules will lead to different points in time when the giant connected component occurs. The percolation in network construction is successfully delayed by their nonrandom edge selection rule. According to community detection process using LPA in original network *G*_0_, we can construct a new network *G*_1_; *G*_0_ and *G*_1_ have the same vertices. If we consider two vertices with the same label in *G*_0_ as two connected vertices in *G*_1_, then we can transform label propagation process in *G*_0_ into the network construction process in *G*_1_ as follows.

The initial status of the new network *G*_1_ is also *n* isolated vertices because of the unique initial labels in label propagation process. For any vertex *i*, denote its neighbor set in original network *G*_0_ by *N*_*i*0_. In *G*_1_, vertex *i* chooses a vertex as its new neighbor from *N*_*i*0_ according to the following rules.

**Rule 1 .**

If there are no connected vertices from *N*_*i*0_, and there is no edge between *i* and any vertex from *N*_*i*0_, we add an edge between *i* and a vertex chosen from *N*_*i*0_ randomly.

**Rule 2 .**

If there are no connected vertices from *N*_*i*0_, and there is an edge between *i* and a vertex from *N*_*i*0_, we delete the edge between *i* and the vertex and add an edge between *i* and a vertex chosen from *N*_*i*0_ randomly.

**Rule 3 .**

If there exist one or more groups of connected vertices from *N*_*i*0_, and there is no edge between *i* and any vertex from *N*_*i*0_, we add an edge between *i* and a vertex randomly chosen from the largest one in these groups (the largest group). When the largest group is not unique, we first choose one randomly from these largest groups.

**Rule 4 .**

If there exist one or more groups of connected vertices from *N*_*i*0_, and there are one or more edges between *i* and the vertices from the unique largest group, we will not add or delete any edge.

**Rule 5 .**

If there exist one or more groups of connected vertices from *N*_*i*0_, and there is an edge between *i* and some isolated vertex from *N*_*i*0_, we delete the edge between *i* and the vertex and add an edge between *i* and a vertex chosen from the largest group randomly. When the largest group is not unique, we first choose one randomly from these largest groups.

**Rule 6 .**

If there exist one or more groups of connected vertices from *N*_*i*0_, and there are one or more edges between *i* and the vertices which are not from the unique largest group, we delete the edges between *i* and this group. If this group is not connected after deleting those edges, we add edges between the vertices in this group to make it connected again and then add an edge between *i* and a vertex chosen from the largest group randomly.

**Figure 2:**
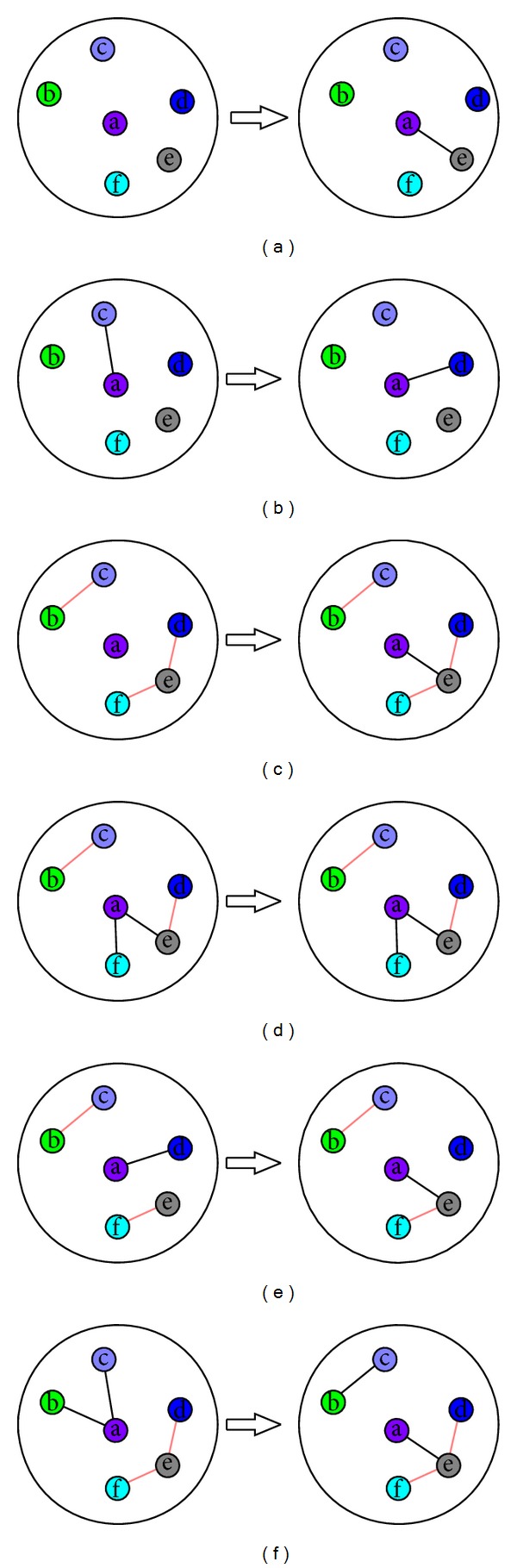
An illustration of network construction rules.

Figure 2 shows an illustration of the six network construction rules. The six vertices in the circle are vertex a and its neighbors b, c, d, e, and f in original network. A red line between two vertices indicates that the two vertices are connected in the new network, and a black line indicates that the two vertices are neighbors in the new network. In every subfigure, the circle on the left shows the current connection status of the six vertices in new network, and the circle on the right shows the new connection status of the six vertices after adding, deleting, or keeping edges in new network.

In Figures 2(a) and 2(b), which explain Rules 1 and 2, the connected vertices in the set {b, c, d, e, f} in new network do not exist, and this means the labels of the five vertices are different in original network. Thus in Figure 2(a) we randomly choose a vertex e from {b, c, d, e, f} and then add an edge between a and e in new network. This means we randomly choose the label of vertex e in a's neighbors as the vertex's new label in original network. In Figure 2(b) there is an edge between a and c, which indicates that a's current label is the same as c's in original network. According to label propagation principle, we randomly choose the label of vertex d in a's neighbors as a's new label in original network again. Thus we delete the edge between a and c and randomly add an edge between a and d in new network.

Figures 2(c) and 2(d) explain Rules 3 and 4. When there are two groups of connected vertices, {b, c} and {d, e, f}, in new network, there exist two groups of a's neighbors with the same labels, {b, c} and {d, e, f}, in original network. According to label propagation principle, we choose a's maximal label as a's new label in original network. Thus in Figure 2(c) we randomly choose a vertex e from {d, e, f} and then add an edge between a and e in new network. In Figure 2(d) there are two edges between a and the unique largest group {d, e, f}, which indicates that a's current label is the same as the label of {d, e, f} in original network. According to label propagation principle, a's label will not change in original network. Thus we will not add or delete any edge in new network.

Figure 2(e) explains Rule 5. When there are an isolated vertex and two groups of connected vertices, {b, c} and {e, f}, in new network, there exist two groups of a's neighbors with the same labels, {b, c} and {e, f}, in original network. Vertex d's label is the same as a's current label, which is different from the labels of b and c, and it is also different from the labels of e and f in original network. According to label propagation principle, when there are multiple maximal labels, we choose randomly one as a's new label in original network. Thus in Figure 2(e) we delete the edge between a and d, choose randomly {e, f} in two connected group, and then add an edge between a and e which is chosen from {e, f} randomly.

Figure 2(f) explains Rule 6. When there are two groups of connected vertices, {b, c} and {d, e, f}, in new network, there exist two groups of a's neighbors with the same labels, {b, c} and {d, e, f}, in original network. We should add an edge between a and a vertex e chosen randomly from the largest group {d, e, f} in new network. But there exist edges between a and {b, c} in new network, which indicates that a's current label is the same as the label of b and c in original network. Thus we delete these edges and add an edge between b and c to make b and c connected in new network again, which means b and c have the same label in original network. Then we add an edge between a and e in new network.

According to these rules, the edges in *G*_1_ will be added, deleted, or kept in a random order iteratively until every vertex has a neighbor in *G*_1_. For a given *G*_0_ we can obtain multiple *G*_1_, but these “*G*_1_” have the same connectivity. If *G*_1_ is a connected graph, all the vertices in *G*_0_ have the same label. Hence, a trivial solution obtained by LPA can be represented by a connected graph obtained by network construction process, and a monster community in LPA can be represented by a giant connected component.

**Figure 3:**
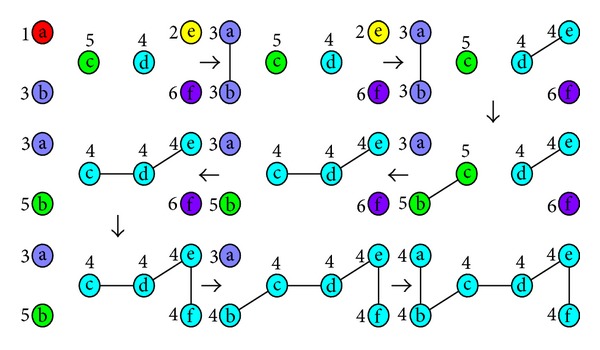
Network construction process.

The occurrence process of monster community in Figure 1 can be transformed into the network construction process in Figure 3.

**Figure 4:**
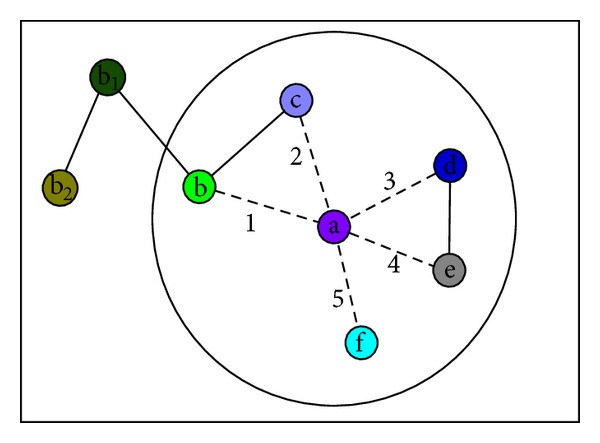
An example for edge selection rules.

In the construction process of several different random graphs, the probability of the giant connected component occurrence is nearly zero when the number of edges is less than *n*/2, but it will increase rapidly when the more edges are added [26, 27]. The larger the current largest connected component, the earlier the giant connected component emergence and the earlier the connected graph formation. Accordingly, we need to avoid adding those edges which will lead to a larger connected component at earlier iterative steps. In the network construction process transformed by label propagation process, the process of adding edges is not random, and the rule of adding edges encourages the formation of a strong local community. As we discussed in Section 2, changing the process greatly is not good for community detection. When there are multiple optimal edges according to the rule of LPA, local communities are not enough encouraged in LPA, which is reflected in the random selection of multiple maximal labels. Thus we should strengthen local communities and constrain their expansion in LPAp. We will show the edge selection rules in Figure 4.

As shown in Figure 4, the neighbors of vertex a are vertices b, c, d, e, and f. Vertices b_1_ and b_2_ are not vertex a's neighbors. In the network, the edge between a and b, the edge between b and c, the edge between d and e, the edge between b_1_ and b_2_, and the edge between b and b_1_ have been added. When vertex a chooses the vertex it will connect to, it can keep the edge 1, or cancel edge 1, and then add edges 2, 3, or 4. When the rule of adding edges is from LPA, vertex a will choose one of the edges 1, 2, 3, and 4 randomly regardless of the label status on the outside of the circle. If vertex a chooses edge 1 or 2, a connected component whose size is 5 will form; if vertex a chooses edge 3 or 4, a connected component whose size is 3 will form. Intuitively, the probability of a giant component occurrence when vertex a chooses edge 1 or 2 is greater than the probability when vertex a chooses edge 3 or 4. Accordingly, choosing edge 3 or 4 can strengthen local community and constrain its expansion.

**Figure 5:**
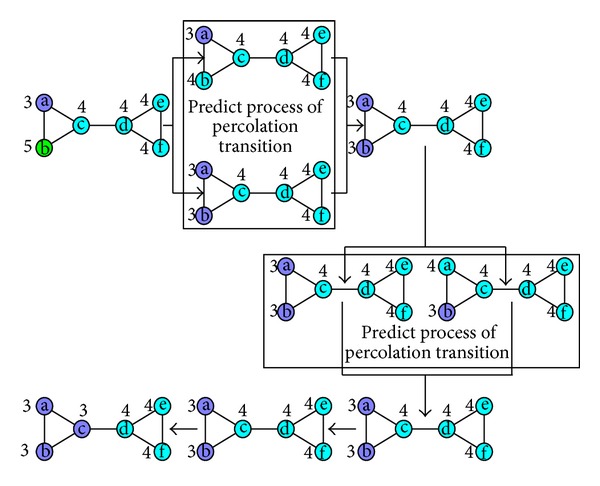
Label propagation with prediction process.

Hence, we add the prediction of percolation transition to delay the occurrence of monster community in label propagation from iteration 2. When there are multiple maximal labels *l*_*i*_^*M*1^, *l*_*i*_^*M*2^,…, *l*_*i*_^*MT*^, we give a vector *S*_*i*_^*M*^ = (*S*_*i*_^*M*1^, *S*_*i*_^*M*2^,…, *S*_*i*_^*MT*^) to record the sizes of new communities for every maximal label after propagating its label. Suppose that *S*_*i*_^*Mt*^ is the minimum element of *S*_*i*_^*M*^, and then we choose the label *l*_*i*_^*Mt*^ as the label of vertex *i*. In Figure 5 we show that how the added prediction process stops the occurrence of monster communities in the same update sequence b-a-f-c-e-d at iteration 2.

As shown in Figure 5, due to the prediction process when the label of vertex b is updated, the algorithm predicts the sizes of potential communities after b's label updating: 5 and 2. Then the algorithm chooses 3 as b's label. So is it when the label of vertex a is updated. We cannot infer that prediction process can stop the occurrence of monster community, but it does delay that.

## 4. Incomplete Update Condition

The added prediction process will make the running time longer, so we give an optional strategy to save time. A vertex can be obviously within a community because its so many neighbors' labels are the same as its label. Leung et al. give the concept of neighbor purity of unweighted networks to measure this property of vertices [12]. Denote the neighbor set of vertex *i* by *N*_*i*_; then we can give the definition of *i*'s neighbor purity:

(12) $$\begin{array}{c} P_{Ni}=\frac{\sum_{j\in N_{i}}\delta \left(l_{i},l_{j}\right)}{\left|N_{i}\right|}, \end{array}$$

where |·| is the measure of a set.

According to the neighbor purity of vertices, Leung et al. try to incompletely update the labels from iteration 2 to save the running time [12]. They only update the labels of vertices whose neighbor purities are greater than a preset threshold. But exchange of a shorter running time is a lower modularity. The modularity after incomplete update may decrease by 30% [12]. The reason is that only using neighbor purity as the criterion of incomplete update, the labels of small degree vertices could not be updated from the earlier iterative steps. The incomplete update condition ignores the contribution of small degree vertices to the whole community structure and performance.

We first generalize the definition of neighbor purity to weighted networks. For a vertex *i*, its neighbor purity can be given by

(13) $$\begin{array}{c} P_{Ni}=\frac{\sum_{j\in N_{i}}w_{ij}\cdot \delta \left(l_{i},l_{j}\right)}{k_{i}}. \end{array}$$

Now we consider the small degree vertices in incomplete update condition. Denote the mean degree of the network by *k*_0_; the incomplete update condition can be given by

(14) $$\begin{array}{c} P_{Ni}\cdot \text{Sgn}\left(k_{i}-k_{0}\right)\geq \varepsilon , \end{array}$$

where *ε* is a preset threshold.

For a given vertex *i*, if incomplete update condition (14) is satisfied, then its label will not be updated. When *i* is a small degree vertex, *k*_*i*_ − *k*_0_ < 0, its label will be updated until the global termination condition is satisfied.

## 5. Algorithm Description

For a given, weighted, and undirected network of *n* vertices with an adjacency matrix *A* ^*w*^, LPAp can be described in the following steps.

1. Initialize the labels of vertices in the network: give every vertex *i* a unique label *l*_*i*_ randomly.
2. Set the number of iterations *k* = 1.
3. Generate a random number permutation *G* ^(*k*)^ without repetition from 1 to *n* and set an update mark *j* = 1.
4. According to the sequence in *G* ^(*k*)^, for every vertex *i*, find out the maximal label *l*_*i*_^*M*^.
5. If *k* > 2, continue; else, go to (7).
6. If *P*_*Ni*_ · Sgn(*k*_*i*_ − *k*_0_) ≥ *ε*, *j* = *j* + 1, and go to (11); else, continue.
7. If there is one maximal label *l*_*i*_^*M*^, go to (10); else, continue.
8. Calculate the size vector *S*_*i*_^*M*^ and find out the minimum *S*_*i*_^*Mt*^ of *S*_*i*_^*M*^.
9. Let *l*_*i*_^*M*^ = *l*_*i*_^*Mt*^.
10. Let *l*_*i*_ = *l*_*i*_^*M*^, and *j* = *j* + 1.
11. If *j* = *n*, continue; else, go to (4).
12. If, for  all  *i*, we have the termination condition, continue; else, set *k* = *k* + 1, and go to (3).
13. Run a breath-first search to separate the communities which are disconnected but with the same label [10].

In the LPAp, the termination condition is still a condition; thus the community structure may be still not unique. The ability to find some communities which cannot be found by some fixed algorithm remains due to its randomness. What we have done is reducing the probability of trivial solutions and keeping near-linear time complexity of the algorithm.

## 6. Experiments

In this section, we carry out community detection on synthetic networks and real-world networks to verify the performance of LPAp, using a tablet PC with 4 GB RAM and a 1.7 GHz dual-core processor running MATLAB 2012b.

### 6.1. Synthetic Networks

LPAp is evaluated from three aspects: the sensitivity of detecting small community, the ability to identify single community structure, and the effect of incomplete update on the speed of community detection and the quality of network division [28].

**Figure 6:**
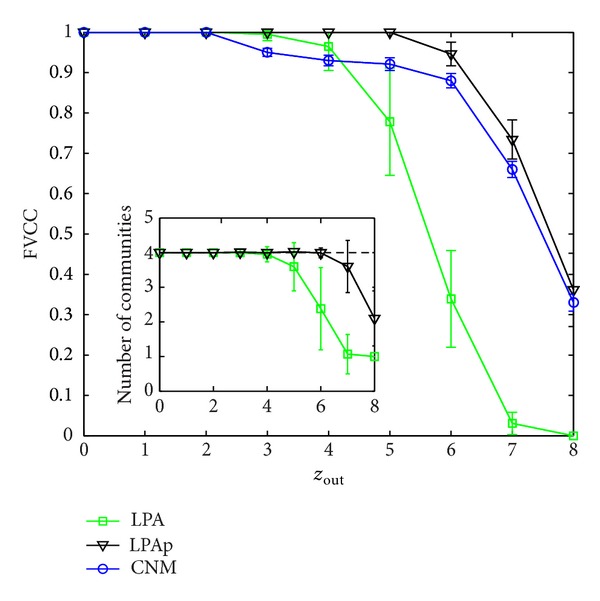
FVCC of CNM, LPA, and LPAp on unweighted computer-generated networks.

The most commonly used synthetic networks are computer-generated networks with 128 vertices, composed of 4 communities with 32 vertices to verify the performance of the algorithms for community detection [6, 29]. The degree of each vertex is 16 on average. Denote the mean number of edges between different communities by *z*_out_. Set the threshold *ε* = 1. For each *z*_out_, we first run the LPAp on ten computer-generated networks and give the results of LPA and CNM for comparisons as near-linear time algorithms. When the number of communities obtained by LPA or LPAp is 1, the algorithm obtains a monster community. The fraction of vertices classified correctly (FVCC) is usually used as accuracy measure of an algorithm on the networks whose community structures are preset or known [6, 13]. In Figure 6 we give FVCC by CNM, LPA, and LPAp, and the numbers of communities obtained by LPA and LPAp are given. Due to the randomness in the three algorithms, every algorithm will run ten times on each network, and each point we will show is an average. Error bars in Figure 6 indicate standard deviations.

As shown in Figure 6, the community structure becomes fuzzy gradually until the whole network is identified as a single community by LPA when *z*_out_ increases. The process in LPAp is delayed and more communities are obtained, which makes LPAp more accurate than LPA and CNM.

**Figure 7:**
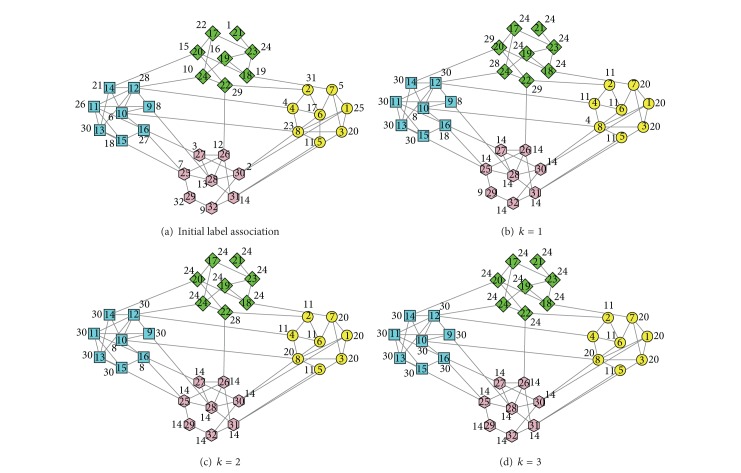
A community detection process on an unweighted computer-generated network using LPAp.

In Figure 7 we give a community detection process on an unweighted computer-generated network using LPAp. For the sake of clarity, the network in this case is a 32-vertex version, where *z*_out_ = 1, and the degree of each vertex is 4 on average. It is designed to consist of four 8-vertex communities. The vertices from the same community are given the same shape and the same color. The number in a vertex indicates the number of the vertex, and the number near a vertex indicates its label. The label update sequences of vertices, which are randomly generated, are 29-16-20-7-10-28-24-14-31-26-27-11-25-8-19-18-21-17-30-6-22-1-2-3-4-13-9-5-32-12-23-15 at iteration 1,6-31-15-5-29-7-20-18-22-11-16-1-10-32-19-24-17-27-9-2-13-14-4-23-3-25-12-21-8-30-28-26 at iteration 2, and 19-1-12-17-23-25-4-3-5-7-8-18-24-13-26-10-15-14-28-30-29-6-31-22-21-9-2-27-16-32-11-20 at iteration 3. In Figure 7 we use *k* to indicate the iterative step.

As we can see, the network consists of four groups of vertices. The edges between the vertices 1–8, the edges between the vertices 9–16, the edges between the vertices 17–24, and the edges between the vertices 25–32 are denser than the edges between the four groups. We run LPAp on it to detect the four communities. After vertex 16's label is updated to 30 at iteration 3, the algorithm is terminated. LPAp identifies three communities exactly and subdivides the fourth 8-vertex community into two 4-vertex communities. The subdivision of a community is considered as right division using FVCC as an accuracy measure, so FVCC by LPAp is 100% in this case. The modularity of this result is 0.50, which is a little lower than the modularity of real division −0.52.

Our algorithm is applicable to weighted networks; thus we give the weighted version of computer-generated networks by assigning each edge a random weight in [0,1]. The weight is random for each edge. For each *z*_out_, using this weighting method on the ten used unweighted networks, we obtain ten weighted computer-generated networks [30]. CNM is an algorithm which is applicable to weighted networks, and LPA has been generalized to weighted networks, so they can be used for comparison [6, 11].

**Figure 8:**
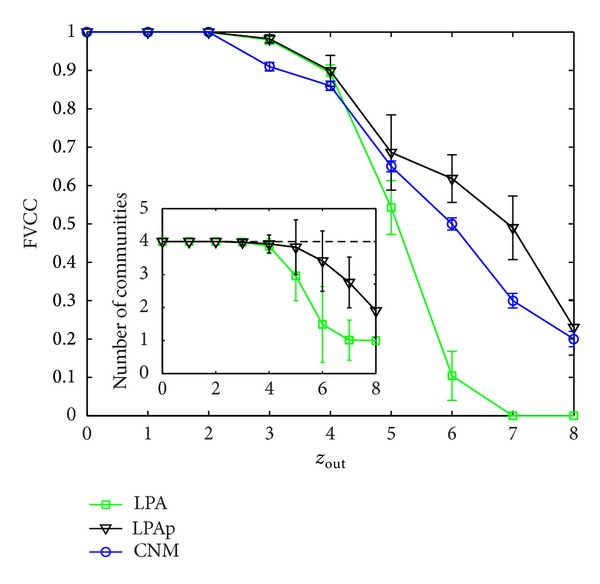
FVCC of CNM, WLPA, and LPAp on weighted computer-generated networks.

In Figure 8, we give FVCC by the three algorithms and the number of communities obtained by WLPA and LPAp. We run each algorithm ten times for each network, and each result is still an average. Error bars in Figure 8 indicate standard deviations.

As shown in Figure 8, when *z*_out_ increases, the numbers of communities decrease gradually and FVCC accuracies of the three algorithms decrease. We get the similar results to the results of the unweighted case. When *z*_out_ = 7, the LPA has identified the network as a monster community. The random assignment of different weights breaks the preset community structures; thus the FVCC accuracies of these algorithms on weighted networks are usually lower than their results on unweighted networks, respectively.

**Figure 9:**
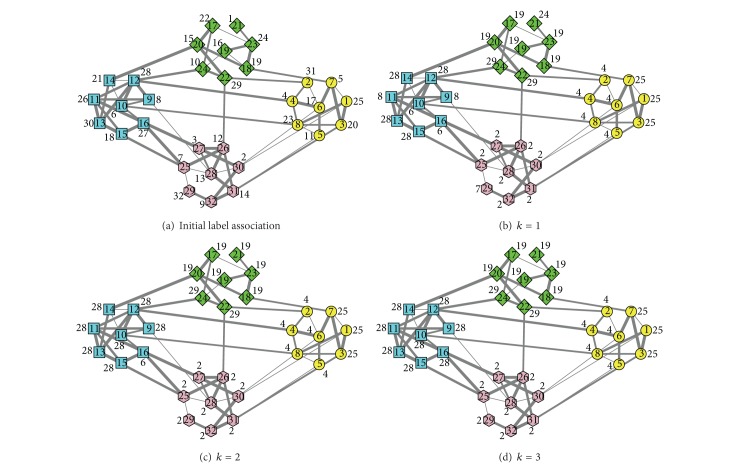
A community detection process on a weighted computer-generated network using LPAp.

In Figure 9 we give a community detection process on a weighted computer-generated network using LPAp. Weigh every edge of the network in Figure 7 using 1, 2, 3, or 4 randomly to obtain its weighted version. To make it intuitive, the widths of lines indicate the weights of edges. The widths 1–4 represent the edge weights 1–4, respectively. For the sake of comparison, the initial label of every vertex and the label update sequences of vertices are the same as the labels and the sequences in Figure 7. The vertices from the same community are given the same shape and the same color. The number in a vertex indicates the number of the vertex, and the number near a vertex indicates its label.

As shown in Figure 9, the community structures in weighted network are not as clear as the community structures in unweighted network. We run LPAp to detect the four preset communities. After vertex 16's label is updated to 28 at iteration 3, the algorithm is terminated. LPAp identifies two communities exactly and subdivides two 8-vertex communities into four smaller communities. FVCC by LPAp is still 100% in this case. The modularity of this result is 0.49, which is much lower than the modularity of real division −0.54. As mentioned in [11], different weights assigned randomly may break the equilibrium within a community or mix different community structures, which makes communities harder to find.

LPA and WLPA tend to find monster community, and this is the reason why their FVCC accuracies decrease so fast. By adding the prediction process in LPAp we can delay the occurrence of monster community, and small communities are easier to be detected. That is the reason why the result of LPAp is more stable though the standard deviations of LPAp are a little larger than CNM's.

An efficient algorithm should not divide a single community network into more communities though the algorithm may tend to find small community structures. We use three kinds of networks to verify the ability of LPAp to identify single community structure. The first one is complete graph. This network is a typical single community structure. The second one is the ER random graph (ER model) whose mean degree is 4. It is not connected, and we use its giant connected component [26]. The last one is computer-generated network (CG network) when *z*_out_ = 8, which has four fuzzy communities. The number of communities detected by an algorithm on these networks can indicate its capability of identifying single community. In order to make the results more intuitive, the sizes of complete graph and ER random models are preset as 128.

In the second column and the third column of Table 1, we give the numbers of communities, respectively, using LPA and LPAp on one complete graph, ten ER random models, and ten computer-generated networks. In the fourth column and the fifth column of Table 1, we give the numbers of communities, respectively, using WLPA and LPAp on one weighted complete graph, ten weighted ER random models, and ten weighted computer-generated networks. The weighting method is assigning each edge a random weight in [0, 1] and the weight is random for each edge. The threshold *ε* is still preset as 1. We run every algorithm ten times for every network. The data in Table 1 are presented as the form *X* ± *Y*, where *X* is the mean number of communities and *Y* is the standard deviation of the number of communities.

As shown in Table 1, the numbers of communities obtained by LPAp are much closer to the actual number than LPA's. On an ER random model the number of communities obtained by LPAp always changes strongly because the communities are not strong enough and these random networks are different. As mentioned in [5], the ER random model whose edges are spare tends to have many small communities. On ER random model, LPAp tends to identify many small communities while LPA and WLPA always obtain a trivial solution. Though LPAp is more sensitive in detecting small communities, the results of LPAp are still the real result 1 for complete graphs. This phenomenon shows that LPAp has still the ability to identify single community, though the network has been weighed. Communities of weighted ER models and CG networks are slightly different from their unweighted versions. That is because the assignment of random weight changes the structures of networks.

To evaluate the effect of incomplete update on the speed of community detection and the quality of network division, we give three different versions of LPAp.

1. LPAp without incomplete update condition: we set *ε* = 1 at (6) of LPAp in Section 5 to make the vertices update their label completely and denote the version of LPAp by LPAp-c.
2. LPAp with incomplete update condition which only considers neighbor purity: we change the condition *P*_*Ni*_ · Sgn(*k*_*i*_ − *k*_0_) ≥ *ε* into *P*_*Ni*_ ≥ *ε* at (6) of LPAp in Section 5 to make all the vertices update their label incompletely irrespective of their degrees and denote the version of LPAp by LPAp-N.
3. LPAp with incomplete update condition which considers both neighbor purity and vertex degree: it is the same as LPAp in Section 5, and we denote it by LPAp-k in light of incomplete condition where vertex degree is considered.

We run LPA, LPAp-c, LPAp-N, and LPA-k on ten used unweighted computer-generated networks when *z*_out_ = 5 and then run WLPA, LPAp-c, LPAp-N, and LPA-k on ten used weighted computer-generated networks when *z*_out_ = 5. In Table 2 we list the running time *t* (in seconds), the mean modularity value *Q*_avg_, and the standard deviation *δ* of the modularity value by these algorithms, respectively, when *ε* = 0.5.

As shown in Table 2, the added prediction process does use extra time, while the mean modularity increases significantly. The incomplete update condition which only considers neighbor purity brings a remarkable decrease of running time, with a lower modularity than LPA or WLPA.

Denote the running time of LPA/WLPA and LPAp-k by *t*_0_ and *t*_*p*_ and the modularity of network division using LPA/WLPA and LPAp-k by *Q*_0_ and *Q*_*p*_. We give *δ*_*t*_ and *δ*_*Q*_ to measure the effect of incomplete update condition on running time and the quality of network division:

(15) $$\begin{array}{c} \delta_{t}=\frac{t_{0}-t_{p}}{t_{p}}\times 100\%,\\ \delta_{Q}=\frac{Q_{p}-Q_{0}}{Q_{p}}\times 100\%. \end{array}$$

**Figure 10:**
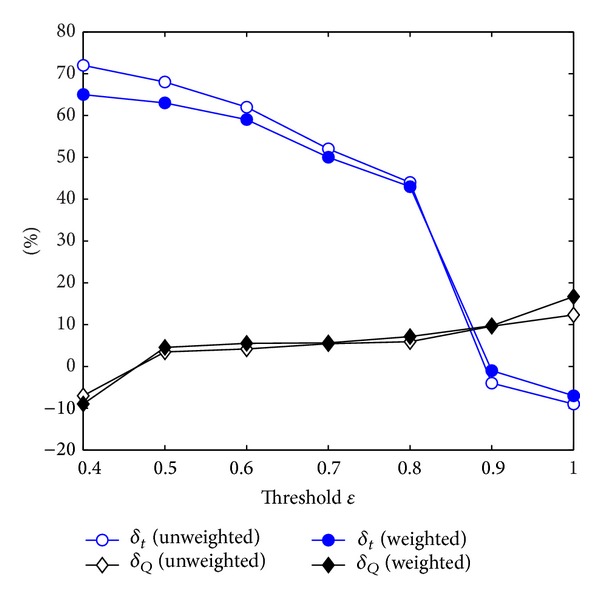
*δ*_*t*_ and *δ*_*Q*_ for different thresholds on unweighted and weighted computer-generated networks.

In Figure 10 we give *δ*_*t*_ and *δ*_*Q*_ for different incomplete update thresholds and *k*_0_ = 16 on ten used unweighted computer-generated networks and ten used weighted computer-generated networks with *z*_out_ = 5. Each point we will show is an average, and the error bars are smaller than the points.

As shown in Figure 10, when *ε* > 0.5, LPAp-k is more accurate than LPA and WLPA. In incomplete update process invalid propagation of labels is avoided and that brings a significant decrease in running time. It is worth noting that when *ε* = 1, LPAp-k is LPAp-c and it uses a little extra time to obtain a much higher mean modularity. Thus the incomplete update process is not a thing we have to do because LPAp-c has done well in accuracy and speed. Certainly, if we need a faster algorithm, incomplete update process will make LPAp-k less time-consuming.

### 6.2. Real-World Networks

There are two ways to verify the performance of a new algorithm for community detection. One is running it on synthetic networks such as those networks in Section 6.1, and the other is running it on real-world networks. In Table 3 we describe six commonly used real-world networks and their basic properties.

In the six real-world networks in Table 3, the club network, the football network, and the dolphin network are usually used for showing the concrete results of community detection as the networks whose community structures are known. The weighted versions of the club network and football network are commonly used too.

In Zachary's karate club network, 34 vertices represent the members of a university karate club, and 78 edges represent their social relations. There are two real communities in club network. In US college football network, the vertices represent college teams, and an edge between two vertices indicates there is one or more games between the two teams during the regular seasons in 2000 [2]. There are 11 conferences and 5 independent teams as real communities of football network. In the weighted football network, there are three edges whose weights are 2 in the 613 edges.

At first, we run LPAp and LPA on unweighted club network ten times and run LPAp and WLPA on weighted club network ten times. In Table 4 we give the maximal modularity, the mean modularity, and the standard deviation of modularity obtained by these algorithms on unweighted version and weighted version of club network, respectively.

As shown in Table 4, the modularity of solutions by LPAp is more stable than LPA's, and the mean modularity of LPAp's solutions is higher than LPA's.

**Figure 11:**
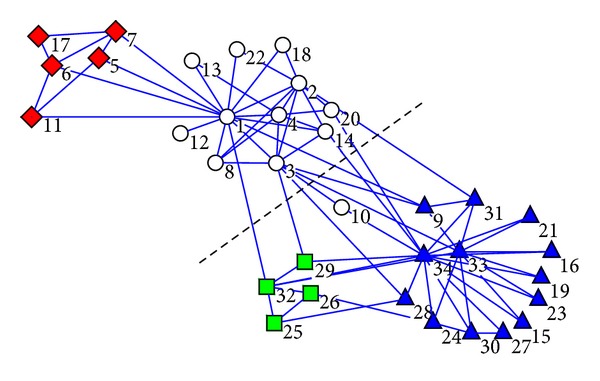
Communities obtained by LPAp on unweighted club network.

In Figure 11 we also give the graphical communities by LPAp on unweighted club network as a concrete result. The vertices which are divided into the same community are given the same shape and the same color in Figure 11. The dotted line in the middle provides a real division of the club network.

Though the controversial vertex 10 is classified wrongly, the modularity *Q* of the solution in Figure 11 is 0.42, which is much higher than 0.37—the modularity of its real community structure.

Next we will execute the same algorithms on unweighted football network and weighted football network ten times, and in Table 5 we give the maximal modularity, the mean modularity, and the standard deviation of modularity obtained by these algorithms on unweighted version and weighted version of football network, respectively.

In Table 5 we get a similar result to Table 4, and the solutions by LPAp are more stable and accurate than LPA's. That is because the prediction process delays the occurrence of monster community which will lead to a low mean modularity.

**Figure 12:**
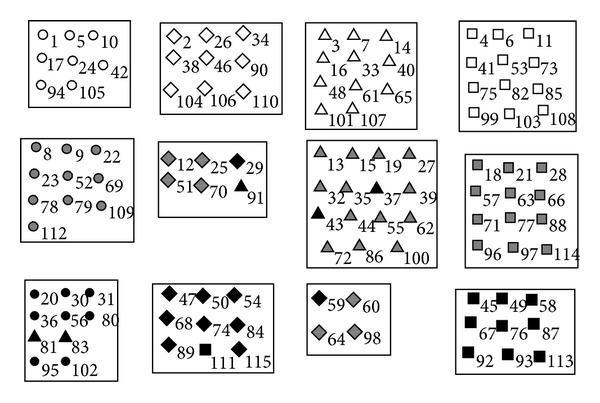
Communities obtained by LPAp on unweighted football network.

In Figure 12 we also show a concrete result of LPAp on unweighted football network. The vertices with the same color and same shape are from the same conference, and the vertices in the same box are divided into the same community by the algorithm.

The modularity of the solution shown in Figure 12 is 0.60, which is higher than 0.55—the modularity of its real community structure. The graphical result of LPAp is the same as the result we get by WLPA in [11]. However, we can get the result by directly running LPAp several times without aggregate, while the same result is obtained by aggregate of multiple results using WLPA for many times.

Finally, we compare FVCC with Girvan-Newman algorithm (GN), LPA, the algorithm using eigenvectors of matrices (EV), spin glass model (SG), LPAm, and LPAp on three real-world networks whose community structures are known in Table 6 and list the mean modularity *Q* and number of communities *N*_*c*_ by these algorithms on six real-world networks for comparison in Table 7 [2, 4, 5, 9, 10, 15, 16]. Due to the randomness in LPA, LPAm, and LPAp, each result of these algorithms is an average over ten runs for each network.

As shown in Tables 6 and 7, though the quality of network division by LPAp is a little shy of the modularity for SG, it is still higher than the modularity by most other algorithms, such as CNM, EV, and LPAm. Moreover, FVCC by LPAp is as high as SG. It is worth noting that SG is a complex algorithm whose time complexity is *O*(*n* ^3^), while LPAp is a near-linear time algorithm.

## 7. Conclusions

In this paper we propose a label propagation algorithm with prediction of percolation transition and incomplete condition to detect community structures in complex networks. The algorithm keeps the near-linear time complexity. To compare with LPA, LPAp is more stable, more efficient, and faster if necessary. The following features of the algorithm can be demonstrated.

1. By considering label status as edge status between two vertices, the community detection process can be transformed into network construction process. Accordingly, the occurrence of monster community in community detection can be delayed by delaying a giant connected component in network construction process.
2. Delaying the occurrence of an uninteresting monster community will provide more opportunity to normal community; thus adding prediction process makes LPAp more sensitive in small community and the ability to identify a single community is maintained.
3. LPAp with incomplete update condition, where the contribution of small degree vertices is considered, can make the computation time be shortened to less than 2/3 of the original label propagation algorithm, and there is nearly no modularity loss.

## Figures and Tables

**Table 1:** The numbers of communities obtained by LPA, WLPA, and LPAp on three kinds of networks.

Network	Unweighted		Weighted	
	LPA	LPAp	WLPA	LPAp
Complete graph	1.0 ± 0.0	1.0 ± 0.0	1.0 ± 0.0	1.0 ± 0.0
ER model	1.0 ± 0.0	45.7 ± 23.9	1.0 ± 0.0	37.4 ± 24.4
CG network	1.0 ± 0.0	2.1 ± 1.2	1.0 ± 0.0	1.9 ± 1.1

**Table 2:** The experimental result of LPA, LPAp-c, LPAp-N, and LPAp-k on computer-generated networks.

Algorithm	Unweighted			Weighted		
	*Q*_avg_	**δ**	*t *	*Q*_avg_	**δ**	*t*
LPA/WLPA	0.38	0.09	0.39	0.35	0.08	0.40
LPAp-c	0.43	0.02	0.43	0.42	0.02	0.42
LPAp-N	0.35	0.03	0.22	0.33	0.04	0.23
LPAp-k	0.38	0.03	0.23	0.37	0.02	0.24

**Table 3:** Description of six real-world networks.

Network	Vertex	Edge	Description
Club	34	78	Relation in karate club [20]
Dolphin	62	160	Dolphin association [21]
Football	115	613	Games between US college football teams [2]
Jazz	198	2742	Collaborations between jazz musicians [22]
*C. elegans *	453	2025	Metabolic reactions in *Caenorhabditis elegans* [23]
E-mail	1133	5451	E-mail contacts at a university [24]

**Table 4:** Comparisons between LPA, WLPA, and LPAp on unweighted and weighted club networks.

Network version	LPA/WLPA			LPAp		
	*Q*_max_	*Q*_avg_	**δ**	*Q*_max_	*Q*_avg_	**δ**
Unweighted	0.40	0.34	0.09	0.42	0.39	0.02
Weighted	0.44	0.39	0.10	0.44	0.43	0.01

**Table 5:** Comparisons between LPA, WLPA, and LPAp on unweighted and weighted football network.

Network version	LPA/WLPA			LPAp		
	*Q*_max_	*Q*_avg_	**δ**	*Q*_max_	*Q*_avg_	**δ**
Unweighted	0.60	0.55	0.07	0.60	0.59	0.01
Weighted	0.60	0.58	0.02	0.61	0.60	0.01

**Table 6:** Comparison between FVCC (%) by different algorithms on three real-world networks.

Algorithm	Club	Football	Dolphin
GN	97.06	83.48	98.39
CNM	97.06	63.48	96.77
LPA	96.47	61.75	83.23
EV	97.06	86.26	98.39
SG	97.06	83.48	98.39
LPAm	97.06	83.48	96.77
LPAp	97.06	83.48	98.39

**Table 7:** Comparison between the results of network division by different algorithms on six real-world networks.

Network	GN		CNM		LPA		EV		SG		LPAm		LPAp	
	*Q *	*N*_*c*_	*Q *	N_c_	*Q *	N_c_	*Q *	N_c_	*Q *	N_c_	*Q *	N_c_	*Q *	N_c_
Club	0.40	5.0	0.38	3	0.34	3	0.38	5	0.42	4	0.35	4	0.39	4
Football	0.60	10.0	0.56	6.0	0.55	10	0.45	13	0.60	10	0.58	10	0.60	12
Jazz	0.41	39.0	0.44	4.0	0.28	2	0.35	8	0.44	5	0.44	4	0.44	5
*C.elegans *	0.40	38.0	0.39	9.0	0.21	8	0.32	28	0.44	10	0.38	9	0.40	10
E-mail	0.53	61.0	0.49	16.0	0.23	4	0.42	45	0.58	12	0.50	10	0.51	17
Dolphin	0.52	5.0	0.50	4.0	0.51	4	0.49	6	0.53	5	0.50	5	0.52	5
